# Lactic Acid: A Novel Signaling Molecule in Early Pregnancy?

**DOI:** 10.3389/fimmu.2020.00279

**Published:** 2020-02-27

**Authors:** Li-Na Ma, Xiao-Bo Huang, Kahindo P. Muyayalo, Gil Mor, Ai-Hua Liao

**Affiliations:** ^1^Institute of Reproductive Health, Tongji Medical College, Huazhong University of Science and Technology, Wuhan, China; ^2^C.S. Mott Center for Human Growth and Development, Wayne State University School of Medicine, Detroit, MI, United States

**Keywords:** lactic acid, pregnancy, aerobic glycolysis, blastocysts, maternal-fetal interface, immune cells

## Abstract

Aerobic glycolysis is a recognized feature shared by tumors, leading to the accumulation of lactic acid in their local microenvironments. Like the tumors, the blastocysts, placenta, trophoblasts and decidual immune cells can also produce a large amount of lactic acid through aerobic glycolysis during the early pregnancy. Moreover, the placenta expresses the transporters of the lactic acid. While several studies have described the role of lactic acid in the tumor microenvironment, especially lactic acid's modulation of immune cells, the role of lactic acid produced during pregnancy is still unclear. In this paper, we reviewed the scientific evidence detailing the effects of lactic acid in the tumor microenvironment. Based on the influence of the lactic acid on immune cells and tumors, we proposed that lactic acid released in the unique uterine environment could have similar effects on the trophoblast cells and immune cells during the early pregnancy.

## Introduction

During embryo implantation, enhanced glucose catabolism leading to the accumulation of lactic acid in the extracellular microenvironment has become a recognized metabolic feature of mammalian blastocysts (1). Indeed, more than 50% of the glucose consumed is not oxidized but is converted to lactate in the presence of oxygen in mouse and human blastocysts (2, 3). Described for the first time by Warburg (4), the formation of lactate from glucose in the presence of oxygen is called aerobic glycolysis or the Warburg effect. Aerobic glycolysis was thought, initially, to be specific to certain cancers, where it generates energy and supplies intermediates for macromolecule biosynthesis pathways (5). Later, the Warburg effect was also found to be a characteristic of other rapidly proliferating cells, such as lymphocytes (6). Note that enhanced aerobic glycolysis does not mean that aerobic oxidation and the tricarboxylic acid (TCA) cycle are completely blocked. However, in aerobic glycolysis, pyruvate is converted to lactic acid by lactate dehydrogenase A (LDHA) (5).

In the tumor microenvironment, the lactic acid produced by aerobic glycolysis is not only used for energy generation but also for other functions. Indeed, lactic acid was described as a key signaling molecule that plays a pivotal role in tumor cell migration, invasion, growth, angiogenesis, and immune escape (7).

A high level of aerobic glycolysis is one of the common characteristics shared by tumors and mammalian blastocysts (1, 5), since blastocysts are also made of rapidly proliferating cells. Indeed, during pregnancy, a high amount of lactic acid is also produced and released at the maternal-fetal interface (1). Although the different functions of lactic acid in the tumor microenvironment are well-studied, unfortunately, its role at the maternal-fetal interface is still not well-investigated.

In this paper, we speculate the current knowledge related to the aerobic production of lactic acid during pregnancy and the scientific evidence describing the effects of lactic acid in the tumor microenvironment. Based on the effects of lactic acid produced by tumors on the local microenvironment, we speculate that lactic acid may have similar effects at the maternal-fetal interface, notably promoting trophoblastic invasion, trophoblastic migration, and maternal angiogenesis; providing metabolic fuel; and modulating decidual immune cells to ensure local immune tolerance and defense against any pathogens (8, 9).

## Lactic Acid

Glycolysis can be defined as the sequence of reactions that breakdown glucose (a 6-carbon molecule) to two molecules of pyruvate. When the oxygen supply is sufficient, pyruvate is converted into acetyl-CoA, which enters the TCA cycle and generates much more ATP. However, under oxygen deprivation conditions, most pyruvate is converted into lactic acid, a process that produces a small amount of energy (10, 11).

Lactic acid, also called 2-hydroxypropionic acid, is a metabolic product of glycolysis that can be produced by most of the tissues in the human body (12). There are two forms of lactic acid, D-lactic acid and L-lactic acid (13). Both forms (stereoisomers) of lactic acid are produced from and metabolized to pyruvate by the action of lactate dehydrogenase (LDH) (14). However, the enzyme is isomer-specific so that production and metabolism of D-lactic acid requires D-LDH and L-lactic acid requires L-LDH. Since human (mammalian) cells only contain L-LDH, in humans the lactic acid produced is almost exclusively L-lactic acid. However, carbohydrate-fermenting bacterial species have by contrast both enzymes and therefore the capacity to produce both D-lactic acid and L-lactic acid (14, 15).

The concentration of lactic acid in human and mouse serum while in a resting state is 1–3 mM but can rise to 15 mM temporarily during strenuous exercise (16). In cerebral ischemia, the lactic acid level can be maintained at 5–10 mM (10). In some tumors, it can reach 20–30 mM in the local microenvironment (17). During embryonic development, blastocysts produce a large amount of lactic acid even in the presence of oxygen, around the time of implantation the mouse and human blastocyst will conservatively consume around 50 to 320 pmols glucose/embryo/h, respectively, the 90% of the consumed glucose forms lactic acid (1).

Recent studies have found that lactic acid plays a key role in multiple cellular processes, including energy regulation, immune tolerance, memory formation, wound healing, ischemic tissue injury, and cancer growth (18).

## Lactic Acid Receptors

When cells use glycolysis, the lactic acid produced in the cells needs to be transported to the outside of the cells to avoid an intracellular accumulation that causes negative-feedback inhibition of glycolytic flux (19). The cells using lactic acid as their energy substrate need to transport extracellular lactic acid into the cell (8). Lactic acid function depends on the specific receptors, primarily monocarboxylic acid transporters (MCTs) and G protein-coupled receptors (GPR81) (20, 21).

### Monocarboxylic Acid Transporters (MCTs)

Lactate is carried across cell membranes by a class of transmembrane proteins known as MCTs [also known as solute carrier 16 (SLC16)], which cotransport protons and lactate anions down the concentration gradients of the lactate anion and proton (22). MCTs constitute a family of 14 transporters, and the most common types are MCT1 (SLC16A1) and MCT4 (SLC16A3) (23).

MCT1 is expressed in tumor cells and is a high-affinity transporter of lactic acid (24). Its main function is to transport extracellular lactate into the cell and thus plays an active role in the uptake of lactate that enters the TCA cycle to increase the energy supply (25). However, under hypoxic conditions, MCT1 can also mediate lactate export (26).

MCT4 is primarily expressed in highly glycolytic cells and is a low-affinity transporter of lactate (27). It is responsible for exporting lactate out of the cell in response to high glycolytic flux and is upregulated in response to hypoxia. For example, under hypoxic conditions, hypoxia-inducible factor 1α (HIF-1α) directly upregulates the expression of MCT4 to promote lactic acid export (28).

### G Protein-Coupled Receptor 81 (GPR81)

GPR81 is also known as hydroxycarboxylic acid receptor 1 (HCA1 or HCAR1) and is expressed in the adipose tissue, skeletal muscle, and brain (20, 29). GPR81 is highly expressed in most tumors, including those in the pancreas, colon, liver, breast, lung, and cervix) (30, 31). GPR81 has been found to be a selective receptor for lactate. Physiological concentrations of lactate are sufficient to activate GPR81, and activated GPR81 downregulates intracellular cAMP levels (18). The lactate/GPR81 pathway plays a key role in multiple cellular processes and functions, including regulating metabolism, such as lipolysis inhibition in adipocytes (32); tumor survival, metastasis and angiogenesis; and immunity (30, 33).

## The Role of Lactic Acid in the Tumor Microenvironment

In tumors, the formation of lactic acid contributes to the acidification in the local tumor environment (34). Moreover, the acidification at the extracellular space is associated with the tumor survival and growth (35). Here, we describe the role of lactic acid in the tumor microenvironment.

### Lactic Acid as an Energy Substrate for Cellular Metabolism

Lactic acid is more than a mere byproduct of glycolysis. Indeed, some recent studies revealed that lactic acid can be a primary source of carbon for the TCA cycle and can be used as an energy substrate in cellular metabolism (36–38). In microenvironments where tumor cells use lactic acid to meet their metabolic needs, for example, in hypoxic regions, the tumor cells release the metabolite lactic acid through MCT4 and recycle it through MCT1 on tumor cells in the oxygenated regions (19, 39). Aerobic cells will then convert lactate into pyruvate to fuel their oxidative phosphorylation, thereby sparing available glucose to reach the hypoxic cells (19).

### Lactic Acid Promotes Tumor Cells Migration and Invasion

In glioblastoma cells, lactic acid enhances matrix metalloproteinase-2 (MMP2) levels by upregulating transforming growth factor β2 (TGF-β2) expression and promotes the migration of glioblastoma cells (9). The high concentration of lactic acid in the tumor microenvironment is associated with distant metastasis and poor prognosis in head and neck cancer and colorectal cancer (40, 41).

MCT-mediated lactic acid flux also helps tumor cells migrate and invade (19). MCT1/MCT4 expression is correlated with tumor progression, tumor recurrence, and decreased patient survival (42, 43). MCT1/MCT4 constitute prognostic markers for poor clinical outcome (44). Blocking MCT1/MCT4 expression impairs tumor cell growth (31).

### Lactic Acid Promotes Angiogenesis in the Tumor Microenvironment

**Figure 1:**
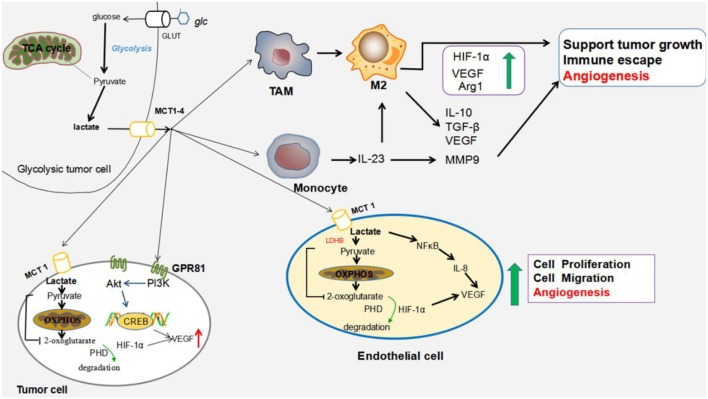
Impact of lactic acid on tumor angiogenesis. Tumor cells generate large amounts of lactic acid that are exported by MCT1 and MCT4. The accumulation of lactic acid in the extracellular milieu promotes several cancer processes leading to cell survival, tumor growth and metastasis. Lactic acid stimulates angiogenesis by polarizing TAMs to the M2-like phenotype; inducing the expression of high levels of VEGF and arginase I (Arg1) that support tumor growth, metastasis, and angiogenesis; and inhibiting antitumor immunity (45, 46); Within monocytes, lactic acid enhances the production of IL-23 (47), and IL-23 stimulates macrophages to produce IL-10, TGF-β, and VEGF (48). IL-23 also induces MMP9 expression, which contributes to angiogenesis. Within endothelial cells, lactic acid activates the NF-κB pathway, which triggers the production of proangiogenic IL-8, drives the migration of endothelial cells and increases the capacity of endothelial cells to form tubes (44). Within the tumor cells, lactic acid activates GPR81 to enhance the secretion of AREG, a member of the EGFR family. AREG contributes to neovascularization by increasing the production of VEGF, which promotes angiogenesis (30, 31). Lactic acid also supports the activation of normoxic HIF-1α in tumors and endothelial cells by inhibiting PHDs with 2-oxoglutarate (49), resulting in the increased expression of relevant pro-angiogenic targets, including VEGF (50). Abbreviations: LDHB, lactate dehydrogenase B; PI3K, phosphatidylinositol 3-kinase; Akt, serine/threonine kinase; TAM, tumor-associated macrophage; VEGF, vascular endothelial growth factor; Arg1, arginase 1; MMP9, matrix metalloprotease 9; AREG, amphiregulin; EGFR, epidermal growth factor receptor; IL-23, interleukin-23; NF-κB, nuclear factor-kappa B; IL-8, interleukin-8; HIF-1α, hypoxia-inducible factor 1α; and PHDs, prolyl hydroxylases.

Lactic acid activates the signaling that promotes angiogenesis. The lactic acid released by cancer cells is notably recognized as an angiogenic promoter. Lactic acid participates in angiogenesis through several mechanisms: activation of GPR81, activation of signaling pathways (HIF-1α and vascular endothelial growth factor (VEGF)/vascular endothelial growth factor receptor 2 (VEGFR2), promotion of cytokine production, induction of Mϕ polarization, and stimulation of endothelial cells. The proposed mechanisms are shown in Figure 1.

### Effects of Lactic Acid on Immune Cells

Lactic acid produced by tumor cells generates an acidic microenvironment (pH 6.0–6.5) (34). That environment promotes tumor survival and growth by directly mediating immunosuppressive effects via a reduction in cytotoxic T cell proliferation and functions; effects on natural killer (NK) cells function, monocyte function and dendritic cell maturation; and skewing of macrophage polarization and cytokine production (45, 51).

#### Cells and NK Cells

**Figure 2:**
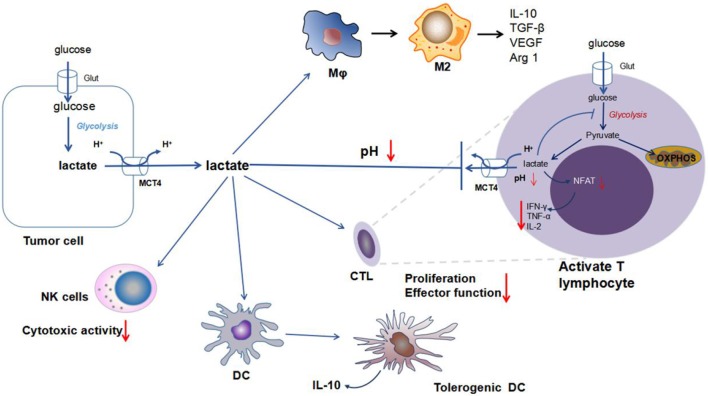
Impact of lactic acid on immune cells in the tumor microenvironment. Enhanced secretion of lactic acid by tumors induces a drop in the extracellular pH and acidification of the tumor microenvironment. There are multiple immune cells in the tumor microenvironment, including TAMs, CTLs, DCs, and NK cells. Increased extracellular concentrations of lactic acid play an important role in tumor escape of immune surveillance. (a), Within activated T cells, a high concentration of extracellular lactic acid from the tumors inhibits the release of lactic acid from T cells. The accumulation of intracellular lactic acid in T cells inhibits glycolysis metabolism and blocks the production of cytokines (particularly IFN-γ, TNF-α, and IL-2). (b), Within macrophages, lactic acid promotes the M2-like polarization of TAMs, generates anti-inflammatory cytokines, such as IL-10 and TGF-β, and increases the production of VEGF and Arg1 for tumor angiogenesis. (c), Within dendritic cells, lactic acid is responsible for the differentiation of tolerogenic DCs and the generation of IL-10. (d), Within NK cells, lactic acid impairs the cytotoxic activity of NK cells. Abbreviations: MCT, monocarboxylic acid transporter; Glut, glucose transporter; NFAT, nuclear factor of activated T cells; TAM, tumor-associated macrophage; CTL, cytotoxic T lymphocyte; VEGF, vascular endothelial growth factor; and Arg1, arginase 1.

It is known that activated T cells and NK cells produce lactic acid by aerobic glycolysis. However, the export of lactic acid relies on the concentration gradient of lactic acid between the extracellular and intracellular environments. Extracellular accumulation of tumor-derived lactic acid inhibits the excrete of lactic acid from the activated T cells, while intracellular accumulation of lactic acid in T cells decelerates energy metabolism and ultimately induces a suppressed phenotype (Figure 2) (45, 51).

Lactic acid accumulation in the microenvironment of tumors inhibits local immunity. For example, lactic acid derived from the tumors inhibits the differentiation of monocytes and T cells *in vitro* (51, 52). Some studies showed that acidosis leads to the loss of tumor-infiltrating T lymphocytes in humans and mice, and T cell function can be restored by adjusting the pH to its normal physiological value (51, 52). Fischer et al. (51) reported that lactic acid decreased the cytotoxic activity of human cytotoxic T lymphocytes (CTLs) by inhibiting their proliferation and cytokine (IL-2 and IFN-γ) production. Lactic acid accumulation in melanoma promotes tumor immune escape and growth by inhibiting the secretion of IFN-γ by effector T cells and NK cells (Figure 2) (45). In CD8^+^ T cells, lactic acid causes the loss of cytolytic functions (53). In addition, lactic acid preferentially enhanced the expression of Th2 cytokine genes (IL-4 and IL-13) but not Th1 cytokine genes (54).

Lactic acid has also been reported to be involved in the immune inflammatory response. For example, lactic acid produced by the tumors promotes the IL-23/IL-17 proinflammatory pathway when monocytes/macrophages were stimulated with a TLR2/4 ligand (47). Lactic acid accumulation leads to an increase in IL-17A production by effector/memory CD4^+^ T and Th17 cells by the IL-23-dependent and -independent pathways (55). Sodium lactate induces CD4^+^ T cells to differentiate into Th17 cells, which produce a large number of proinflammatory cytokines, such as IL-17 (55). However, the main point is that the definite role of IL-17 in tumorigenesis is uncertain yet (56). Therefore, it needs further confirmation whether the role of tumor-derived lactate on Th17 cells is to promote immune response or to promote tumor immune escape.

Nevertheless, it should be noted that regulatory T (Treg) cells can thrive in a high lactic acid environment, and their proliferation and function are not affected, which play a key immunosuppressive role in tumor microenvironment (57, 58). It is reported that lactic acid can induce large percentage of Foxp3^+^ Treg cells in breast cancer by plasmacytoid dendritic cells and induce NF-kB-mediated Foxp3 activation, hence driving the differentiation of Treg cells from naive CD4^+^ T cells (59). Moreover, the lactate/GPR81 pathway also elicit anti-inflammatory effects. GPR81 mediates the expression of lactate-induced programmed cell death protein-1 (PD-1) ligand (PD-L1) in lung cancer cells. Lactate-induced activation of PD-L1 in tumor cells can reduce the production of IFN-γ and induce the apoptosis of cocultured Jurkat T-cell leukemia cells (60). When GPR81 gene is deleted in mice, Th1/Th17 cell differentiation increases and Treg cells differentiation decreases, resulting in enhanced susceptibility to colon inflammation (61).

#### Dendritic Cells

Dendritic cells (DCs) are important antigen-presenting cells (APCs), these cells link the innate and adaptive immune systems by presenting antigens to T cells and providing the costimulation and cytokines required for the activation of antigen-specific T cells (62).

Studies of the effects of lactic acid on dendritic cells show that monocyte-derived DCs (MoDCs) produce lactic acid, which inhibits CD1a^+^CD142^+^ MoDC differentiation and promotes anti-inflammatory cytokine IL-10 production (63). IL-10 influenced the differentiation and maturation of DCs *in vitro* (64). Moreover, lactic acid produced by the tumors can also inhibit the differentiation of DCs by altering their antigen presentation and functional activity (Figure 2) (65).

#### Macrophages

Macrophages are heterogeneous cell population whose phenotypes are shaped by various microenvironment stimuli. IFN-γ and LPS induce classical activation of macrophages (M1) (66), while IL-4 and IL-13 induce alternative activation in macrophages (M2) (67).

M1 and M2 macrophages differ in their metabolism and immune function (68). M1 macrophages are involved in the defense against bacterial infections and obtain energy through glycolysis, while M2 macrophages are involved in tissue repair and wound healing and utilize oxidative phosphorylation as their main source of energy (66, 68). Energy metabolism is necessary for the correct polarization and function of macrophages (68). Current research shows that macrophages not only play an important role in the host defense against pathogens and the adaptive immune response but can also promote the proliferation and invasion of cancer cells (69). In the tumor microenvironment, tumor-associated macrophages (TAMs) are similar to alternatively activated macrophages (70). The lactic acid produced by the tumors is a signal that induces TAMs to polarize to the M2-like phenotype (71). Major mechanistic studies found that lactic acid promoted the M2-like polarization of TAMs via HIF-1α signaling independent of the IL-4 and IL-13 pathways and induced the expression of high levels of VEGF and arginase 1 (Arg1) (Figure 2) (71). In breast cancer, lactic acid drives M2 macrophage polarization via the activation of the ERK/STAT3 signaling pathway to promote cancer proliferation, migration, and angiogenesis (70). Lactic acid can also activate G protein-coupled receptor 132 (GPR132) on the macrophages to facilitate M2-like polarization (72).

## Aerobic Glycolysis and Lactic Acid Production During Pregnancy

Like the tumor microenvironment, the maternal-fetal interface in the first trimester of pregnancy has similar hypoxic, acidic, and immune features in the microenvironment (34, 73). In the first trimester of pregnancy, maternal blood vessels of the placenta have not been established, the concentration of oxygen within the lumen of the uterus is relatively anoxic. In the early pregnancy, intervillous oxygen tension rises steeply from <20 mmHg (equivalent to 2–3% O_2_) at 8 weeks of gestation to > 50 mmHg (>6% O_2_) at 12 weeks of gestation (74). Lower oxygen concentrations are related to the up-regulation of glucose metabolism and the formation of high levels of lactic acid.

### Blastocysts

When the fertilized egg forms the blastocyst, glucose becomes the main nutrient source. During embryonic development, the 90% of the consumed glucose are not oxidized but instead converted to lactic acid in the presence of oxygen (1). This suggests that a large amount of lactic acid may contribute to the formation of an acidic environment in early pregnancy, which benefits the implantation of the embryo. Indeed, Xiao et al. (75) showed that the acidification of mouse uterus tissue was associated with early uterine preparation for embryo implantation. Therefore, lactic acid may play an important role in regulating acidification of the uterus for the implantation of the embryo (76).

### Placenta

The placenta is an essential organ that provides vital support for oxygen exchange, hormone production, and nutrition flow from the mother to the developing fetus throughout gestation (77). Each step in these processes requires metabolic processes (78). Aerobic glycolysis also exists in the placenta. Glucose is metabolized by the placenta to produce lactic acid, which is the key fuel for fetal growth (78). Approximately 25% of the CO_2_ produced by the fetus comes from the oxidation of lactate that was delivered from the placenta (79).

Healthy placental metabolism is very important for a successful pregnancy (80). Some maternal diseases, including preeclampsia (PE) and gestational diabetes, are related to abnormal placental metabolism (81, 82). One study found that the glycolysis and lactic acid production rates in the placenta of PE patients were significantly lower than those of healthy pregnant women (82). Therefore, understanding the role of lactic acid at the maternal-fetal interface may provide a new direction for the prevention and treatment of pregnancy-related diseases.

### Trophoblasts

After successful implantation and initiation of placentation, trophoblast cells undergo extensive proliferation and differentiation.

There are two main pathways by which trophoblast differentiation may occur, villous and extravillous differentiation (80). Trophoblasts differentiated along the villous pathway include cytotrophoblasts (CTB) and syncytiotrophoblasts (SCT) (83). CTB are located on the basal side of the trophoblasts and face the fetal surface, and SCT are located on the top of the villi facing the maternal surface (83). Glucose is the main carbohydrate source of energy for the trophoblast (80). Kolahi et al. (84) measured the extracellular acidification rate (ECAR), an indicator of aerobic glycolysis, and the oxygen consumption rate (OCR), an indicator of OXPHOS in the trophoblast. Both were greater in CTB than in SCT *in vitro*, which indicates that CTB rather than SCT principally drive placental glucose consumption and lactate production. Kay et al. (85) measured the levels of lactate in culture supernatants from trophoblastic cell line JEG3 under both normoxic and hypoxic conditions (1% oxygen). Their results show that JEG3 cells can produce higher lactate in all time points of 6, 12, and 24 h under hypoxia than normoxia. At 24 h, the level of lactate in the culture supernatant of JEG3 cells is an approximate 2-fold increase under hypoxia. Also, both the mRNA and protein levels of MCT4 (lactate transporter) are significantly increased under hypoxia compared to normoxia. These findings demonstrate that JEG3 cells can actively produce and secret lactate under hypoxic conditions.

### Decidual Stromal Cells

Decidualization is critical for pregnancy, this is a necessary step for successful implantation and placentation (86). Enhanced glucose influx is critical for decidualization (87). One study found that aerobic glycolysis also occurs in mouse decidua and produces a large amount of lactic acid. In decidualizing cells, progesterone activates HIF-1α and c-Myc through the PI3K/Akt signaling pathway to maintain aerobic glycolysis (76). Inhibition of lactate flux leads to compromised decidualization and decelerated lactate-dependent proliferation (76). Zuo et al. (76) suggested that aerobic glycolysis and local lactate flux in decidual cells play important roles in supporting early pregnancy.

### Decidual Immune Cells

Initially, aerobic glycolysis was considered a metabolic pathway unique to tumor cells (4). However, it has been recently shown that aerobic glycolysis is also associated with many other normal cells, such as immune cells. For example, a switch from oxidative phosphorylation to aerobic glycolysis is an important feature of activated macrophages, dendritic cells, and T cells, particularly the Th1 and Th17 subsets (88, 89). Augmented aerobic glycolysis was also found in lymphocyte, and all of these cell populations produce lactate at some point (6).

In summary, the blastocyst, placenta, trophoblast and decidual immune cells may produce a large amount of lactic acid through aerobic glycolysis at the maternal-fetal interface during the first trimester of pregnancy, leading to a decrease in the pH of the extracellular microenvironment. Furthermore, lactic acid also accumulates in the vaginal lumen of healthy reproductive age women to participate in antimicrobial and immune modulatory activities at vaginal lumen and optimizes epithelial cell functions, facilitates prolongation of gestation until term in the pregnancy (90). Unfortunately, the functions of aerobic glycolysis and lactate at the maternal-fetal interface remain insufficiently understood. Based on the influence of the tumor's acidic, hypoxic microenvironment on immune cells and tumors (45, 91), the similar biological behaviors between the trophoblast cells and tumor cell (92), the unique uterine environment could have similar effects on trophoblasts and immune cells at the maternal-fetal interface during the first trimester of pregnancy.

## The Possible Role Of Lactic Acid At The Maternal-Fetal Interface

In the uterus, GPR81 expression is high in the myometrium, increases during gestation, and peaks near labor (93). It is worth noting that the human placenta, like other tissues, expresses many MCT subtypes. Of the MCT family members, MCT1, MCT4, MCT5, MCT6, MCT8, and MCT10 have demonstrated significant mRNA expression in the human placenta (94). MCT1 and MCT4 are localized on the basal plasma membrane and apical microvillous membrane of SCT in the human term placenta (95). Hypoxia promotes the expression of MCT4 in trophoblasts (85). The possible reason for the polarity of MCT1 and MCT4 in the placenta is that the expression of MCT4 in the maternal surface of the SCT promotes the excretion of lactic acid, which facilitates lactate efflux, helps to maintain placental and fetal pH during times of glycolytic stress and prevents lactic acidosis from occurring in the fetal circulation (95). MCT1 is mainly located on the fetal-facing surface and is considered to be important for lactate influx so that lactate can enter energy metabolism pathways (96). These findings suggest that MCTs and GPR81 at the maternal-fetal interface may help lactic acid play certain roles by mediating the signaling and transport of lactic acid.

In pregnancy, lactic acid is an important metabolite used as a metabolic energy source in a variety of fetal tissues, for example, porcine fetal heart (97), ovine fetal liver (98), and rat fetal brain (99). In fetal calves, L-lactic acid is considered to be one of the major carbon sources for fatty acid synthesis (100). In the first trimester, undifferentiated decidual cells import lactic acid through MCT1 to use as an energy source for cellular proliferation, and the inhibition of lactic acid transport causes the failure of decidualization (76).

In humans and other mammals, the level of lactic acid in the fetal circulation is significantly higher than that in the maternal circulation (79, 101), suggesting that L-lactic acid may be an energy substrate for fetal growth.

Decidual immune cells play an important role in maternal-fetal tolerance and embryonic development (102). The immune cells at the maternal-fetal interface mainly include NK cells, macrophages (Mϕ), regulatory T cells and DCs (92). Uterine NK cells and Mϕ are the most prominent decidual immune cells; decidual NK cells account for ~50–70% and decidual macrophages (DM) for 20–25% of all decidual immune cells (103, 104). Decidual NK cells have low cytotoxicity as compared to peripheral blood NK cells (105). The phenotype of DM contain M1 and M2 (106). Although it has been reported that the polarization of DM is regulated by cytokines (IL-4, IL-13, IL-10, and M-CSF, etc.), chemokines, hormones (estrogen, progesterone, HCG, etc.) and signaling pathways (Notch, etc.), but the regulation of macrophage polarization is still unclear (107, 108). During normal pregnancy, Th1/Th2 immunity alterations with a shift to a predominantly Th2-type immunity (109). Furthermore, a decrease in IL-17A production was observed in decidual T cells compared to peripheral blood T cells (110). Pregnant women had high level TGF-β and low level IFN-γ as compared to non-pregnant women (111). Projecting from the effects of lactic acid on immune cells in the tumor microenvironment, lactic acid generated in the first trimester of pregnancy could play an important role in immune regulate functions, potentially by decreasing the cytotoxic activity of T cells and NK cells, promotes Th2-type and regulate the polarization of DM.

Similar to what occurs in the tumor microenvironment, we propose that lactic acid stimulates angiogenesis at the maternal-fetal interface through acting on the macrophage and endothelial cells.

## Conclusion

It is noteworthy that a large amount of lactic acid is produced during embryonic implantation and development. However, the role of lactic acid at the maternal-fetal interface is still unclear. Based on the influence of lactic acid in the local microenvironment of tumors and recognizing the similarities between tumor cells and trophoblasts, we suggest that lactic acid might play a similar role at the maternal-fetal interface. Indeed, during pregnancy, lactic acid could play a role in trophoblast invasion and angiogenesis. Lactic acid might be considered a novel signaling molecule for the modulation of decidual immune cells during the early pregnancy, inducing immune tolerance to allogeneic fetuses and requiring further investigation.

## Author Contributions

All authors listed have made a substantial, direct and intellectual contribution to the work, and approved it for publication.

### Conflict of Interest

The authors declare that the research was conducted in the absence of any commercial or financial relationships that could be construed as a potential conflict of interest.
